# Polyvinyl alcohol film with chlorine dioxide microcapsules can be used for blueberry preservation by slow-release of chlorine dioxide gas

**DOI:** 10.3389/fnut.2023.1177950

**Published:** 2023-04-18

**Authors:** Hongxia Su, Zhanpeng Chen, Yuan Zhao, Jiejie An, Haohe Huang, Ren Liu, Chongxing Huang

**Affiliations:** ^1^School of Light Industry and Food Engineering, Guangxi University, Nanning, China; ^2^Guangxi Key Laboratory of Clean Pulp and Papermaking and Pollution Control, Nanning, China

**Keywords:** chlorine dioxide, microencapsulation, double emulsion, PVA film, blueberry preservation

## Abstract

**Introduction:**

Chlorine dioxide (ClO_2_) is a safe and efficient bactericide with unique advantages in reducing foodborne illnesses, inhibiting microbial growth, and maintaining the nutritional quality of food. However, gaseous ClO_2_ is sensitive to heat, vibration, and light, which limits its application.

**Methods:**

In this study, a ClO_2_ precursor-stabilized ClO_2_ aqueous solution was encapsulated by the double emulsion method, and a high-performance ClO_2_ self-releasing polyvinyl alcohol (PVA) film was prepared to investigate its performance and effect on blueberry quality during storage.

**Results:**

The self-releasing films had the best overall performance when the microcapsule content was 10% as the film's mechanical properties, thermal stability, and film barrier properties were significantly improved. The inhibition rates of Listeria monocytogenes and Escherichia coli were 93.69% and 95.55%, respectively, and the mycelial growth of Staphylococcus griseus was successfully inhibited. The resulting ClO_2_ self-releasing films were used for blueberry preservation, and an experimental study found that the ClO_2_ self-releasing antimicrobial film group delayed the quality decline of blueberries. During the 14-day storage period, no mold contamination was observed in the ClO_2_ self-releasing film group, and blueberries in the antibacterial film group had higher anthocyanin accumulation during the storage period.

**Discussion:**

Research analysis showed that films containing ClO_2_ microcapsules are promising materials for future fruit and vegetable packaging.

## 1. Introduction

Blueberries, also known as lingonberries, belong to the family *Rhododendronaceae*, genus *Lingonberry* and are perennial deciduous or evergreen shrubs. Studies have shown that blueberries are rich in many biologically beneficial compounds, including anthocyanins, glucose, fructose, vitamins (A, D, and E), folic acid, minerals (such as phosphorus, potassium, and magnesium), and organic acids (1, 2), which have high healthcare value and development prospects (3). However, owing to their thin skin and lack of hard shell protection, blueberries are susceptible to mechanical damage during harvest, transportation, and storage (4–6), which leads to the susceptibility of blueberries to microbial infestation after harvest (7, 8). Mold is a common cause of blueberry rot, which affects its quality and reduces the economic benefit of the blueberry industry. Consequently, traditional packaging can no longer meet the high standard of food quality and health requirements. Most blueberry post-harvest diseases are caused by fungi, with various regions having different but related blueberry disease-causing fungi, mainly gray Staphylococcus, Penicillium, and anthropoid polychaetes (9, 10). Post-harvest quality decline and fungal rot have caused huge losses to the economic value of blueberries, greatly limiting the development of the blueberry industry.

With the increase in safety awareness, people have higher packaging requirements (11–15). ClO_2_ is a low-cost, highly efficient, and safe antimicrobial agent, which has strong antimicrobial activity against bacteria, fungi, yeast, and mold, has good application prospects, and is often used as a gas or liquid to preserve the freshness of fruits and vegetables (16). ClO_2_ can penetrate through the cell membrane of microorganisms, destroying their osmotic pressure and oxidizing enzymes containing sulfur groups and some amino acids in proteins. This breakdown and destruction of amino acids, in turn, control the synthesis of microbial proteins. ClO_2_ can interact with bacterial DNA and RNA, eventually leading to bacterial death (17, 18). Additionally, ClO_2_ has good killing effects on budding spores, viruses, algae, and fungi. For example, it has been shown that ClO_2_ can rapidly oxidize and destroy the tyrosine in the virus protein coat, inhibiting specific virus adsorption and preventing host cell infection (19). Owing to its strong oxidizing properties, ClO_2_ not only prevents the decomposition of methionine, but also quickly eliminates ethylene (20). In 2001, the FDA approved for food packaging of raw meat, such as poultry and seafood (21, 22). Mu et al. (23) investigated the effect of combined treatment with ultrasound and ClO_2_ aqueous solution on the post-harvest storage quality of spinach during storage and found that the combined treatment was effective in improving the storage quality of spinach. However, the ClO_2_ solution was unstable, needed to be prepared on-site for use, and could not be stored or transported. Additionally, spraying ClO_2_ solution on fruits and vegetables adds moisture to them, which is not conducive to their subsequent storage. Moreover, because of the irregular surface of fruits and vegetables, ClO_2_ solution cannot completely cover their surface, which limits its application. In contrast, gaseous ClO_2_ has a higher permeability and is therefore preferred. Liu et al. (24) demonstrated that ClO_2_ has a degradation effect on aflatoxin in peanuts and ClO_2_ gas fumigation is more effective than ClO_2_ aqueous solution. However, ClO_2_ itself is unstable and very sensitive to heat, vibration, and light; hence, a volume concentration of more than 10% in the air will be explosive, limiting its application. Therefore, finding ways to stabilize ClO_2_ and ensure sustained release with long-lasting effects on fruit and vegetable preservation is currently a concern.

Microencapsulation involves the formation of a micron-sized core-shell structure by wrapping active ingredients with polymer materials. This process can effectively isolate the external environment from oxygen, light, ions, and other unfavorable factors, which helps enhance the stability of the core material. The application of microencapsulation technology for fruit and vegetable preservation is a crucial innovation. Liu et al. (25) prepared antimicrobial films that spontaneously generated ClO_2_ gas under moisture activation. The slow release of ClO_2_ gas through antimicrobial films can effectively inhibit the growth and reproduction of bacteria and molds on the surface of strawberries, thus extending their shelf life and maintaining their color, hardness, and other sensory characteristics. In addition, many solid ClO_2_ carriers reported in studies are petroleum-based polymers. However, petroleum-based plastics are difficult to degrade in nature, and most of the waste spreads, migrates, and accumulates in the environment and causes great harm to humans and other organisms. Moreover, when incinerated, they cause new environmental pollution problems (26, 27). Polyvinyl alcohol (PVA) is an FDA-approved synthetic polymer known as a “green polymer” because of its good water solubility and relative ease of degradation (28). PVA also has the advantages of non-toxicity, good biocompatibility, and good film formation and is a sustainable alternative to non-biodegradable polymers (29). Miao et al. (30) prepared a novel slow-release active PVA film loaded with tea polyphenol microcapsules, which had excellent antioxidant activity and sustained release and effectively extended the shelf life of food. Antibacterial packaging can reduce or hinder the growth of microorganisms, extend shelf life, and improve product safety (31). In this study, PVA films loaded with chlorine dioxide microcapsules were prepared by a double emulsion method and the performance and effect of a ClO_2_ self-releasing PVA film were investigated on blueberry quality during storage. It has a positive effect on delaying blueberry spoilage and maintaining food safety.

## 2. Materials and methods

### 2.1. Materials and reagents

Cellulose acetate butyrate (CAB), gelatin, and sodium dodecyl sulfate (SDS) were purchased from Shanghai Aladdin Biochemical Technology Co. Aqueous chlorine dioxide solution was purchased from Guangzhou Lightning Biotechnology Co. Ltd., sodium chloride (NaCl) was purchased from Aladdin Reagent (Shanghai) Co. Nutrient agar (NA), nutrient broth (NB) were purchased from Beijing Luqiao Technology Co., Ltd., and potato dextrose agar (PDA) was purchased from Beijing Auboxing Biotechnology Co. All the reagents were used upon receipt.

### 2.2. Preparation of chlorine dioxide microcapsules

ClO_2_ microcapsules were prepared by the double emulsion method. The internal aqueous phase (W_1_) was prepared by weighing 8 wt% of gelatin in a stable aqueous solution of ClO_2_ and stirring in a water bath at 40°C until dissolved. The internal aqueous phase (W_1_) was prepared by weighing 15 wt% of gelatin in a stable aqueous solution of ClO_2_ and stirring until dissolved in a water bath at 40°C; then, 1 wt% of cellulose acetate butyrate and span 80 were weighed into ethyl acetate and dissolved by magnetic stirring to obtain the oil phase (O), with a constant span 80 concentration of 1 wt%. A certain amount of sodium dodecyl sulfate was dissolved in distilled water to obtain the external aqueous phase (W_2_) with a 1 wt% concentration.

At 25°C, a certain amount of the oil phase was weighed into the beaker, and the mechanical stirring (524G) speed was adjusted to 1,000 rpm, after which a certain amount of internal water phase solution was added, and the water-in-oil emulsion was formed after 4 min of stirring. The mechanical stirring was turned off, and the speed was adjusted to 550 rpm by weighing a certain amount of the external aqueous phase solution, then slowly adding the freshly prepared water-in-oil solution to the external aqueous phase. The double emulsion of water-in-oil-in-water was formed by stirring. With the evaporation of ethyl acetate, the double emulsion was cured to form the ClO_2_ microcapsules. The cured microcapsules were washed several times with distilled water to rinse off the unencapsulated ClO_2_ solution on the surface, and then the precipitate was dried under a vacuum to obtain the ClO_2_ microcapsule powder.

### 2.3. Preparation of PVA films loaded with chlorine dioxide microcapsules

A total of 10 wt% of polyvinyl alcohol was added to deionized water at 95°C and dissolved completely to make a 10% concentration of PVA solution. After cooling to room temperature, 5 wt% of boric acid and 0 wt%, 5 wt%, 10 wt%, 15 wt%, and 20 wt% of microcapsules were added and stirred well, and a film-forming solution was obtained after standing and defoaming. The film was coated by a flat coating method to obtain a uniform thickness of the composite films. The roller height of the coating machine was set at 1,000 μm and the speed at 5 mm/s. Antibacterial films of different formulations were obtained by natural drying. All samples were stored in a constant temperature and humidity chamber (LRH-250-HS) of 23°C and 50% RH for 48 h before use.

### 2.4. Characterization of PVA films loaded with chlorine dioxide microcapsules

#### 2.4.1. Apparent appearance

Scanning electron microscopy (SEM) (F16502) was used to observe the morphology of the microcapsule-loaded antimicrobial films. A small amount of sample was added to the surface of the sample stage with conductive double-sided adhesive, the excess sample was blown off, and the sample was sputtered with gold spray for 60 s at an observation voltage of 10 kV before image acquisition. The antimicrobial film was brittle and fractured by liquid nitrogen to obtain the cross-section, and the cross-section was observed using the above procedure.

#### 2.4.2. Fourier infrared (FTIR)

The chemical structures of the antimicrobial films and the possible interactions between their components were identified using attenuated total reflection Fourier transform infrared (ATR-FTIR) (Nicolet iS50) spectroscopy with a Bruker TENSOR, German spectral scan range of 400–4,000 cm^−1^.

#### 2.4.3. X-ray diffraction

X-ray diffraction (A24A10) was used to analyze the physical phase of the PVA films and antimicrobial films with different microcapsule contents. Cu-Kα (=0.154 nm) was used as the emission source to generate the rays with an instrument voltage of 30 kV, tube current of 15 mA, scanning speed of 5°/min, and scanning range of 10–50°.

#### 2.4.4. Thermogravimetry

The thermal stabilities of the PVA and antimicrobial films with different microencapsulation contents were analyzed using a simultaneous thermal analyzer (STA449F5, Germany). The samples were cut to a fine size, weighed approximately (3 mg−8 mg) using an analytical balance, and added to a tared crucible with a programmed temperature rise rate of 10°C/min, a temperature range of 25°C−600°C, and a nitrogen purge of 20 mL/min.

#### 2.4.5. Mechanical properties

The tensile strengths and elongations at the break of the films were determined using an electronic universal tensile tester (AGS-X 100KN). The films were cut to 70 × 15 mm according to the ASTMD882-12 standard; the initial pitch was set to 50 mm, and the speed was set to 50 mm/min. The thicknesses of the PVA and antimicrobial films with different microencapsulation contents were tested using a thickness tester. Ten points were selected for each sample strip, five parallel strips were tested for each group of samples, the average value was obtained, and the relative standard deviation was calculated.

#### 2.4.6. Barrier properties

The ultraviolet-visible (UV-Vis) absorption spectrum of each composite antimicrobial film was recorded in the wavelength range of 200–1,000 nm. The film samples were cut into rectangular strips (10 × 40 mm) and placed in a cuvette, and the absorbance was measured at 600 nm (Agilent 8453). The test was repeated three times for each film, using an empty cuvette without a film as a reference. The opacity was calculated using the following equation (32):


(1) $$\begin{array}{l} Opacity=\frac{Abs600}{t} \end{array}$$

where Abs600 is the absorbance value of the antimicrobial film at 600 nm and t is the thickness of the antimicrobial film (mm).

An oxygen transmission rate tester (GDP-C) was used to test the oxygen permeability of the PVA film and antimicrobial films with different contents. The test area was 5 cm^2^, and three parallel samples were set for each group of samples.

A water vapor transmission rate tester (W3-031) was used to measure the water vapor transmission of PVA film and antimicrobial films with different contents at 38°C, 90% RH, 30 min test interval, and 33.18 cm^2^ test area. Three parallel samples were used for each group.

#### 2.4.7. Antibacterial properties

The method described by Liu et al. (25) was used, with minor modifications, to evaluate the antibacterial properties of the films. Gram-negative and Gram-positive bacteria, represented by *Escherichia coli* and *Listeria monocytogenes*, respectively, were used as bacterial inhibition targets.

At the end of this process, the degree of bacterial growth was expressed as a percentage as follows:


(2) $$\begin{array}{l} R=100\;\times \frac{\left(A\;\times \;B\right)}{A} \end{array}$$

where R is the bacterial inhibition rate (%), A is the total number of bacteria, and B is the number of colonies on the antibacterial film.

The potato dextrose agar (PDA) of weight 46 g and distilled water of 1,000 mL were added, heated until completely dissolved, and autoclaved at a temperature of 115°C for 30 min. PDA medium was poured into a sterile Petri dish and set aside for condensation. The coagulated PDA medium should be completely covered with 0.1 g of antimicrobial film-forming solution. The cakes were cut along the edge of the colony with a sterilized 6-mm diameter punch in a sterile station and placed on the PDA medium covered by the film-forming solution. The dishes were incubated in a constant temperature incubator at 28°C for 5 days, and then the mycelial diameter was measured.

#### 2.4.8. Slow-release properties

The films were placed in a constant temperature and humidity chamber of 23°C and 90% RH, and the ClO_2_ slow-release characteristics were measured using the titration method. Before the test, the films were cut up, with the appropriate amount of potassium iodide and distilled water added, and shaken for 1 h at 37°C and 250 rpm to fully release the ClO_2_ inside the films, which oxidized the potassium iodide to iodine monomers. The amount of ClO_2_ remaining inside the films was subsequently calculated by titration with sodium thiosulfate solution to indirectly obtain the daily released gas ClO_2_. Three parallel samples were available for each test. Each sample weighed 0.8 g.

### 2.5. Blueberry freshness determination

The blueberry variety used in the experiment was Sapphire from Yunnan, which was selected on the same day by the farm and immediately transported to the laboratory. Blueberry fruits of similar shape and size with no obvious mechanical damage were randomly divided into three groups for the freshness test: blank control (CK), pure PVA film, and PVA antimicrobial film with ClO_2_ microcapsules. In the blank control group, blueberries were placed in uncovered plastic boxes without any treatment, and in the pure PVA film and antimicrobial film groups, blueberries were wrapped in bags using a heat-sealing mechanism for freshness preservation. All samples were placed in an artificial climate chamber (LRH-250-HS) with parameters set at 23°C and 90% RH to detect changes in blueberry quality within 2 weeks. Each group was tested three times with a 1-day interval between each test.

#### 2.5.1. Weight loss

Blueberries of similar quality and ripeness were selected for weight loss testing using an analytical balance (EL204), and the weighing results were retained at three decimal places. The test was repeated three times for each group with a day interval between tests.

#### 2.5.2. Textural characteristics

Textural properties of the three groups of blueberries during storage were determined using a TMS-PRO series texture analyzer. Blueberries were tested for TPA by pressing intact blueberry fruits in the center with a flat cylindrical probe of 100 N. The parameters were set to a pre-test speed of 30 mm/min and a time-test speed of 60 mm/min. The parameters of the mass spectrometer were set as follows: 30 mm/min pre-test speed, 60 mm/min test speed, 30% compression deformation, 5 g trigger force, and 5 s interval between compressions. Twenty blueberries were measured in each group, and the average value was recorded. The hardness, elasticity, and chewiness data of the blueberries were obtained from the texture property curves.

#### 2.5.3. Soluble solids (TSS)

The soluble solids were determined using an Abbe refractometer (K7135). Approximately 5 g of blueberry fruit was randomly weighed in each treatment group, ground, and centrifuged at 4,000 rpm for 10 min, and the test was repeated three times in each group with a 1-day interval between each test.

#### 2.5.4. pH

For each treatment group, 50 g of blueberries was weighed and stirred into blueberry homogenate with a juicer, then centrifuged at high speed for 10 min at 4°C. The upper layer of blueberry juice was tested with a pH agent (STARTER 3100), and the test was repeated three times in each group with a 1-day interval between each test.

#### 2.5.5. Chromatic aberration

The surfaces of the blueberries were measured using a spectrophotometer (Agilent 8453), and each group of blueberries was measured three times. The results are expressed as L^*^ and a^*^ values, where L^*^ represents the degree of brightness (value range: 0–100) and a^*^ represents the red and green values. When a^*^ is positive: the larger the value, the greater the degree of redness, and when a^*^ is negative: the larger the value, the greater the degree of greenness.

#### 2.5.6. Anthocyanins

Anthocyanins were determined by spectrophotometry. One gram of blueberry was weighed, a small pre-cooled 1% HCL-methanol solution was added, then the homogenate was ground in an ice bath and transferred to a 20-mL graduated test tube. The mortar was rinsed with 1% HCL-methanol solution and transferred to the test tube, the volume was mixed to scale, and extracted for 20 min at 4°C in the dark; it was shaken several times during the period. The filtrate was collected for use as a blank control. The absorbance values of the filtrate were measured at 600 nm and 530 nm and repeated three times. The difference between the absorbance values at 530 nm and 600 nm was used to express the anthocyanin content, that is, U=(OD530–OD600)/g. Each test was repeated three times with a 1-day interval between each test.

#### 2.5.7. Low-field MRI

A low-field benchtop pulse analyzer was used to measure the effects of moisture distribution and migration throughout the fruit during storage. The samples were placed in the center of a glass cuvette with a diameter of 15 mm. Carr-Purcell Meiboom-Gill (CPMG) sequence scan tests were performed to obtain exponential decay curves, and spin-spin relaxation areas were determined by inversion operations of the curves, which were used to assess the water state and activity. The τ value was 200 μs and the cycle delay was 8,500 ms.

## 3. Results and discussion

### 3.1. Characterization of PVA films loaded with chlorine dioxide microcapsules

Based on the pre-experiments, the optimal ratio for preparing microcapsules was determined to be 8% gelatin, 15% wall material, 7:100 W_1_:O, and 2:1 W_2_:O. The encapsulation rate of microcapsules prepared at this ratio was 74.64%, and the average particle size was 25 μm. Thus, PVA films loaded with chlorine dioxide microcapsules were prepared.

#### 3.1.1. Surface morphology analysis

**Figure 1:**
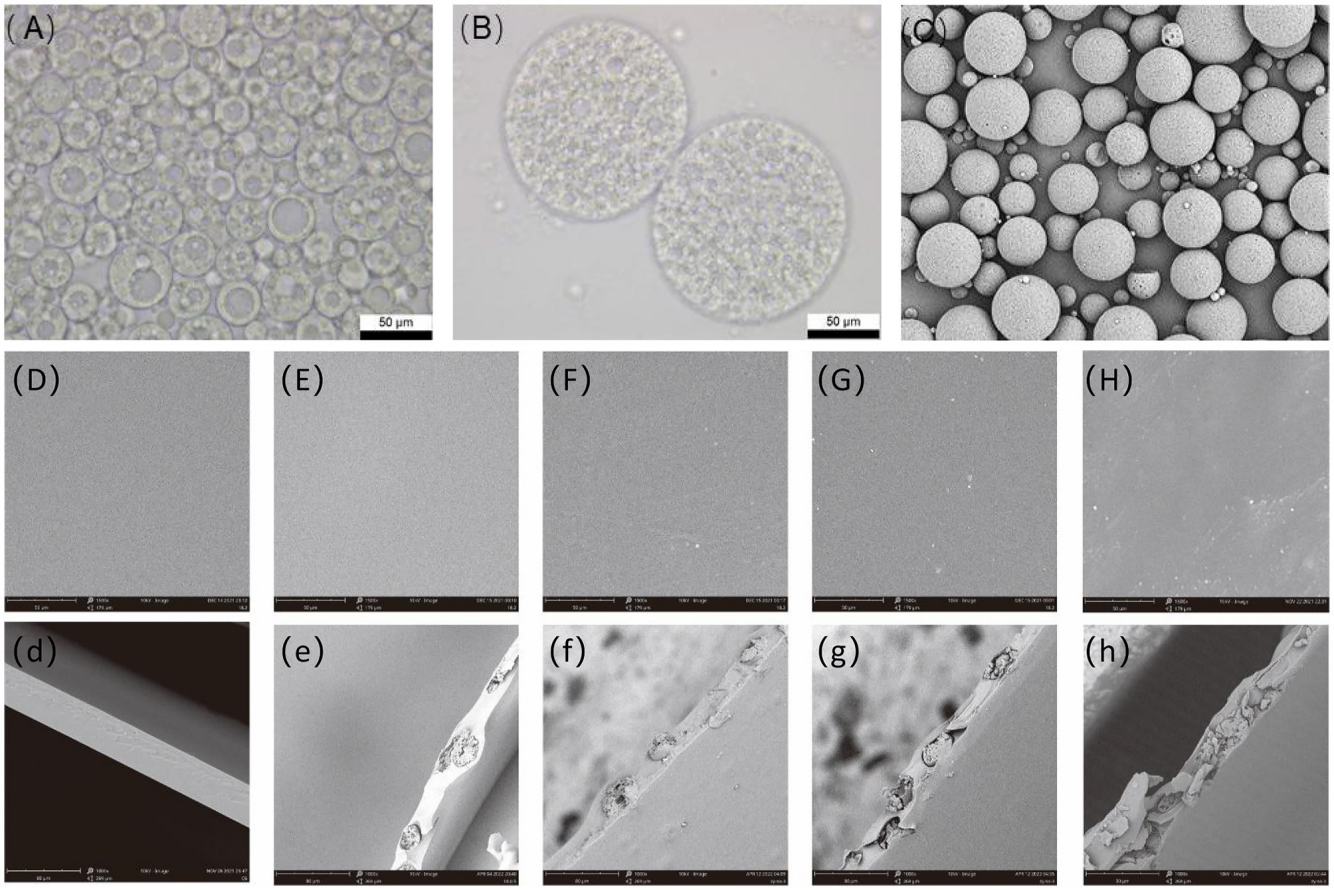
SEM of optical micrograph of the double emulsion under the optimal process **(A, B)**, scanning electron microscopy of microcapsules **(C)**, and antibacterial film with different microcapsule addition amount, **(D)** microcapsule addition amount is 0%, **(E)** microcapsule addition amount is 5%, **(F)** microcapsule addition amount is 10%, **(G)** microcapsule addition amount is 15%, and **(H)** microcapsule addition amount is 20%. SEM, scanning electron microscopy.

Optical microscopy observations (Figures 1A–C) revealed three types of double emulsion droplets: those encapsulating only one internal aqueous phase droplet; those encapsulating several, but no more than 10, internal aqueous phase droplets; and the most complex droplet system encapsulating a large number of internal aqueous phases. SEM also showed that the majority of the prepared double emulsion systems contained a large number of internal aqueous phase droplets, which demonstrated a retarding effect (33). SEM was used to examine the surface of microcapsules prepared by the optimal process. Figure 1C shows that the prepared microcapsules are regularly spherical in shape, with uniform particle size, no adhesion, and a relatively rough surface.

The planes and cross-sections of the pure PVA films and composite films of different proportions were observed, as shown in Figure 1. The prepared thin surface of pure PVA film was smooth, without cracks and voids. An appropriate number of microcapsules were well dispersed in the PVA film and did not affect the appearance of the film. When the microcapsule content was increased to 10%, the film surface remained flat but with spherical protrusions (Figure 1F). When the film contained a large number of microcapsules, the microcapsules were partially agglomerated, increasing the film's roughness and making the surface rough and uneven, with the microcapsules protruding. A comparison of the cross-sectional images revealed that the pure PVA film cross-section was smooth, and microcapsules were observed in the cross-section of the composite film, thus demonstrating the successful encapsulation of microcapsules in the PVA substrate. However, the inhomogeneous dispersion of excessive microcapsules in the antimicrobial film can lead to local agglomeration of microcapsules and disrupt the film structure, drastically affecting the mechanical properties, barrier properties, and thermal stability of the antimicrobial film (25, 34). Therefore, the microencapsulation content within the PVA film must be controlled to prevent excessive negative effects.

#### 3.1.2. Fourier variation infrared spectroscopy and X-ray diffraction analysis

**Figure 2:**
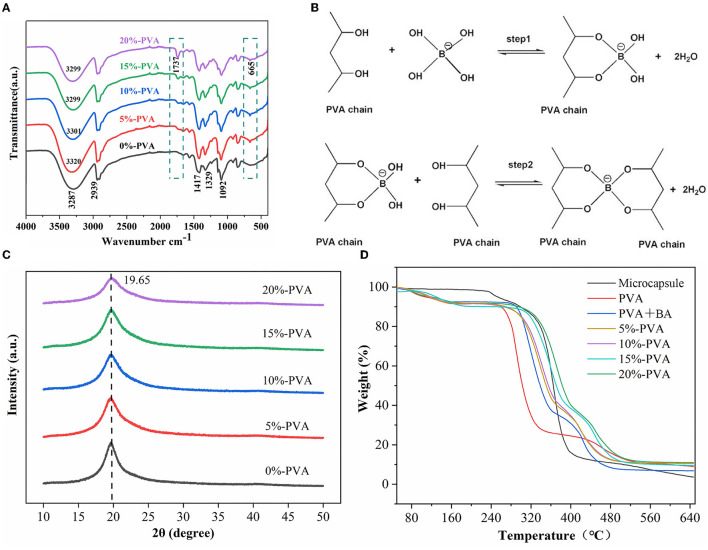
Infrared spectra of PVA films with different microcapsule contents **(A)**, mechanism of cross-linking reaction between PVA and borate ion **(B)**, scanning electron microscopy (XRD) patterns of PVA films with different microcapsule contents **(C)**, and thermal properties of the antibacterial film **(D)**. PVA, polyvinyl alcohol.

The structure of the pure PVA film and the spectral variation of the composite film were characterized using ATR/FT-IR with a spectral resolution of 4 cm^−1^ (Figure 2A). The broadband between 3,000 and 3,500 cm^−1^ centered at 3,187 cm^−1^ in the pure PVA spectrum is attributed to the hydroxyl stretching vibration because there are many hydroxyl groups on the main chain of PVA, which can easily form intra- and intermolecular hydrogen bonds. This band is also characteristic of PVA (35). The band at 2,939 cm^−1^ is generated by C-H stretching (36). Other important peaks of PVA are at 1,417 cm^−1^ and 1,092 cm^−1^, corresponding to the bending stretching of -CH_2_ and -CO (37). The hydroxyl groups on the repeating units of PVA can act as reaction sites and react with other chemicals to cross-link PVA, creating a three-dimensional network and improving its mechanical properties and thermal stability. In this experiment, PVA and boric acid were cross-linked, and the mechanism was considered to be diol-type, with the two diol units of PVA forming a cross-link with a boronic acid ion. The reaction process involves two steps: the mono-diol complexation step and the cross-linking reaction step, as shown in Figure 2B. The typical FT-IR absorption peaks of boric acid at 2,259 cm^−1^ and 1,250 cm^−1^ are not shown in the figure, indicating that boric acid is completely involved in the cross-linking reaction (38). Compared with pure PVA, a small peak was observed at 1,737 cm^−1^ in the composite film, which is because the wall material of the microcapsules is cellulose acetate butyrate, and 1,737 cm^−1^ is the carbonyl characteristic peak of cellulose acetate butyrate. This peak also indicates that the microcapsules were successfully introduced into the film (39, 40). Additionally, the composite film showed a small peak at 665 cm^−1^, attributed to the O-B-O stretching vibration compared to pure PVA. The composite film shows different degrees of red shift in the broad spectrum at 3,000–3,500 cm^−1^ compared to pure PVA, indicating the formation of a cross-linked network of boric acid and PVA (41).

To determine whether microcapsules affect the crystal structure of PVA, we performed scanning electron microscopy tests on the pure and composite PVA films, and the results are shown in Figure 2C. The diffraction spectrum of pure PVA shows a typical crystalline peak at 2θ = 19.65°, corresponding to the (101) plane of semi-crystalline PVA (42). The characteristic diffraction peaks of PVA appeared in the diffraction spectra of all the composites, indicating that introducing microcapsules did not change the crystal structure of PVA (43).

#### 3.1.3. Thermal stability analysis

The thermal stability of the films was tested using a thermogravimetric analyzer. The results are presented in Figure 2D. The pure PVA films showed a typical three-stage thermal degradation process. The first stage occurred below 129°C with a mass loss of 8.03%, mainly due to the volatilization of water; the second stage occurred between 235 and 315°C with a mass loss of 62.08%, mainly due to the degradation of PVA side chains; and the third stage occurred between 422 and 513°C with a mass loss of 10.06%, mainly due to the degradation of PVA's main chain (39). The thermal stability of the composite film was significantly higher than that of the pure PVA film. For example, the film with 10% microencapsulation addition had T10% and T50% values of 274°C and 355°C, respectively, which was a significant improvement compared to the 0%-PVA film, with T10% at 235°C and T50% at 278°C. The thermal decomposition temperature of the hybrid films shifted to a higher temperature range than that of PVA, indicating enhanced thermal stability of the composite films. Liu et al. (25) obtained similar results that a small number of microcapsules can improve the thermal stability of the film. This is due to two factors: First, the wall material of microcapsules, cellulose acetate butyrate, has a higher decomposition temperature than that of PVA; hence, the addition of microcapsules improves the thermal stability of the film. Second, the cross-linking of boric acid and PVA forms a dense network structure that limits the movement of PVA chain segments, also improving the thermal stability of the film, according to the literature (33). These two points are also reflected in the results of our experiments. Therefore, the content of the introduced microcapsules needs to be controlled to prevent degradation of the thermal stability of the films, and similar conclusions were obtained by SEM.

#### 3.1.4. Mechanical property analysis

Food packaging films must maintain integrity to avoid external damage to food during transportation, handling, and storage (44). Table 1 lists the stress–strain curves obtained from the tensile experiments. The pure PVA film exhibited the best flexibility with an elongation at a break of 197%, while its breaking strength was the lowest at only 36.91 MPa. The addition of 5% microcapsules increased the tensile strength of the antibacterial film to 68.89 MPa, which was 1.86 times that of the pure PVA film, indicating that the addition of an appropriate amount of microcapsules improved the tensile strength of the film, and the appropriate microcapsules dispersed in the PVA matrix acted as a nucleating agent. In addition, the elongation at break of the 5% microencapsulated antimicrobial film was only slightly reduced compared to that of the pure PVA film. The cross-linking of boric acid with PVA to form a dense network also improved the film's breaking strength (39, 45). However, the cross-linking effect was a double-edged sword as it limited the sliding of the polymer chains during the tensile test, thus reducing the tensile properties of the film, which is consistent with the behavior exhibited during thermogravimetry. Increasing the content of microcapsules in the antimicrobial film to 10% further decreased the elongation at the break of the antimicrobial film to 124.18%; nonetheless, its breaking strength was only slightly reduced to 66.74 MPa. As the number of microcapsules added was further increased, the microcapsules in the PVA matrix appeared to agglomerate, resulting in a disruption of the antimicrobial film structure. The agglomerated microcapsules caused uneven thickness of the film, resulting in stress defects and a substantial decrease in the composite film's tensile strength and elongation at break.

**Table 1:** Effect of different microcapsule contents on mechanical properties, barrier properties, and antibacterial properties of PVA film.

**Fraction**		**Different microcapsule contents**				
		**0%**	**5%**	**10%**	**15%**	**20%**
Mechanical property	Tensile strength (MPa)	36.91	68.89	66.74	55.03	40.20
	Elongation at break (%)	197	162.85	124.18	70.02	62.18
Barrier property	UVT (%)	88.78	60.32	45.29	32.23	18.69
	WVTR (g·m^−2^·24 h^−1^)	466.76 ± 10	399.36 ± 15	361.18 ± 17	379.39 ± 23	396.59 ± 16
	OTR (cc·m^−2^·24 h^−1^)	69.67 ± 0.6	69.99 ± 0.7	71.98 ± 0.4	75.35 ± 0.3	76.22 ± 0.7

UV transmittance (UVT) is a measurement of the amount of ultraviolet light (commonly at 254 nm due to its germicidal effect) that passes through a water sample compared to the amount of light that passes through a pure water sample; Water Vapor Transmission Rate (WVTR) is the rate at which water vapor will permeate through solid material over a specific period of time; Oxygen Transmission Rate (OTR) is the steady-state rate at which oxygen gas permeates through a film at specified conditions of temperature and relative humidity.

#### 3.1.5. Barrier performance analysis

The transmittance of the films was evaluated in the wavelength range of 200–1,100 nm, and the results are presented in Table 1. The pure PVA film exhibited good transmittance in both the UV and visible ranges. However, the composite film with added microcapsules exhibited significant UV absorption, with the film containing 20% microcapsules having 85.72% lower transmittance at 280 nm compared to the pure PVA. This indicates excellent UV-blocking properties that can effectively resist the deterioration of food due to UV irradiation, thus ensuring food quality during storage and transportation and extending its shelf life (46). Opacity was determined by dividing the absorbance value at 600 nm by the thickness of the film. With increasing microcapsule content, the opacity of the films increased, and the transparency of the composite films with microcapsules decreased significantly. This decrease in light transmission was mainly due to the opacity of the microcapsules dispersed in the polymer matrix, which prevented the passage of light (47). However, the calculated opacity at 20% microcapsule addition was still below 5, indicating that all laminates clearly show package content and status (48).

The water vapor barrier performance and oxygen barrier performance are two important indicators for evaluating food packaging films. If the barrier performance of the packaging film is poor, the packaged food is exposed to a moist environment, which can easily lead to food spoilage. Similarly, oxygen can oxidize the food, which is detrimental to maintaining the quality and safety of the food (44). As shown in Table 1, the H_2_O(g) and O_2_ gas transport rates of PVA films containing ClO_2_ first decreased and then increased with increasing microcapsule content, which is similar to the results reported by Liu et al. (34). The water vapor transmission rate of the pure PVA film was found to be 466.76 g/m^2^·24 h, which gradually decreased with the addition of microcapsules. The water vapor transmission rate reached a minimum of 361.18 g/m^2^·24 h when the microcapsules were added at 10%, which is about 22.61% lower than that of pure PVA film. With the increase of microcapsules, the amount of water vapor transmission also gradually increased, but even 20%-PVA water vapor barrier performance was still better than that of the pure PVA film. This is because boric acid cross-linked with PVA produces a denser network, which prevents water vapor transmission (38). The addition of microcapsules also makes the transport path of water vapor in the film longer and more curved, thereby increasing the difficulty of water vapor transmission (49). However, too many microcapsules form agglomerates, which destroy the film structure resulting in increased water vapor transmission (47). Moreover, as shown in the graph, the composite film maintained the original oxygen barrier performance of PVA, and the oxygen transmission rate fluctuated around 72 cc·m^−2^·24 h^−1^.

#### 3.1.6. Antibacterial performance analysis

**Figure 3:**
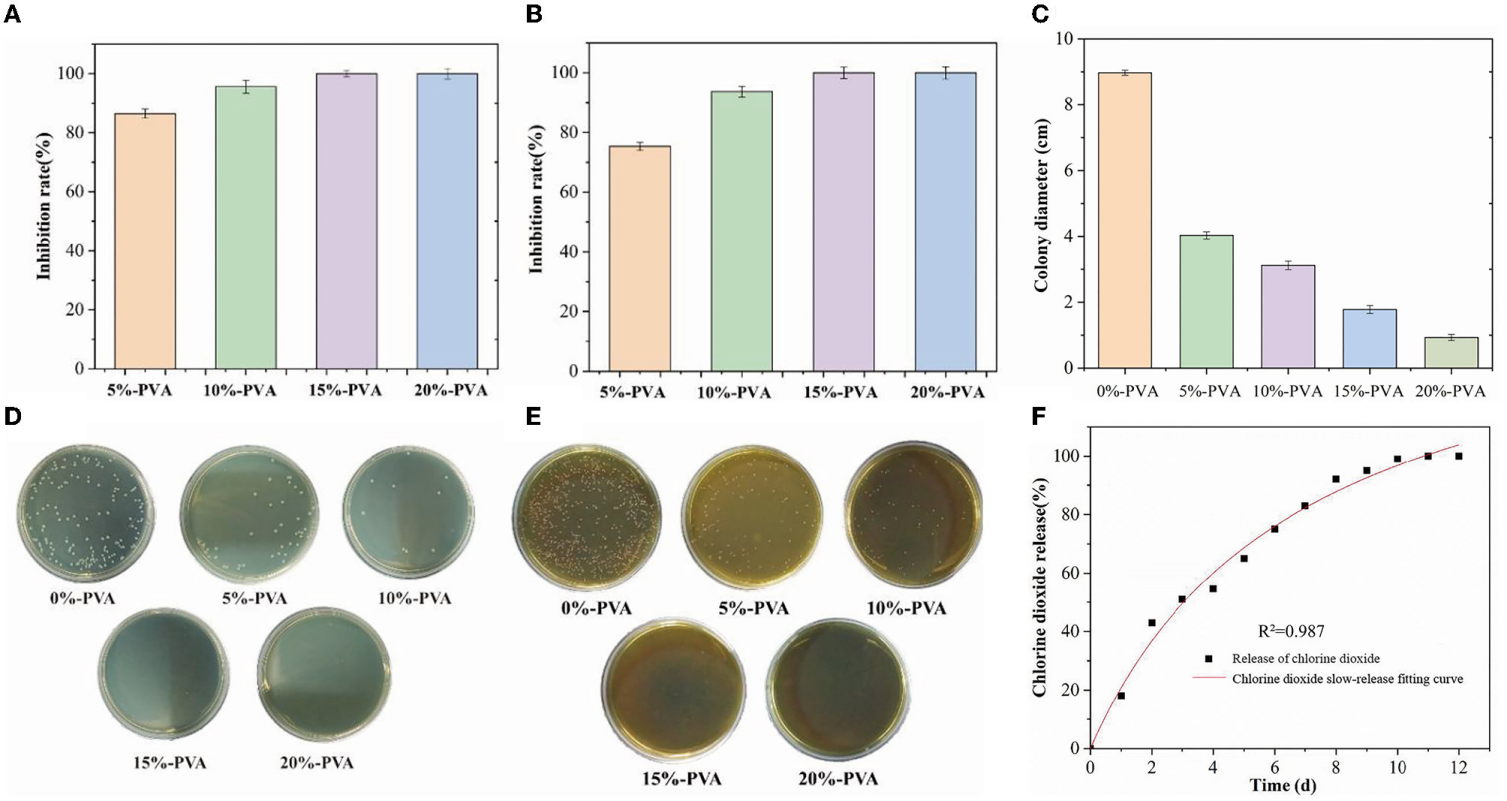
Bacteriostatic effect of PVA films with different microcapsule contents on *Escherichia coli* and *Listeria*, **(A, D)** are *Escherichia coli*, and **(B)** and **(E)** are *Listeria*. Bacteriostatic effect of PVA film with different microcapsule contents on the mold **(C)**, and logistic release model fitting of the antimicrobial film **(F)**. PVA, polyvinyl alcohol.

The microcapsules inside the film react with boric acid to release ClO_2_ gas, which can inhibit bacterial growth. According to the literature, ClO_2_ exhibits efficient and broad-spectrum inhibition properties against both Gram-negative and Gram-positive bacteria (50). We tested the antibacterial performance of pure PVA films and composite films with different microcapsule contents against *E. coli* and *Listeria monocytogenes*, using *E. coli* as a representative of Gram-negative bacteria and *Listeria monocytogenes* as a representative of Gram-positive bacteria, as shown in Figures 3A, B, D, E. The addition of 5% microcapsules showed good antibacterial performance, with an inhibition rate of 86.43% for *E. coli* and 75.32% for *Listeria monocytogenes*. When the microcapsules were added at 10%, the inhibition rate of *E. coli* was 95.55%, and that of *Listeria* monocytogenes was 93.69%. When the addition amount was 15%, the composite film achieved a 99.99% inhibition rate for both *E. coli* and *Listeria monocytogenes*. It is generally believed that ClO_2_ achieves its antibacterial effect through three pathways: (i) rupture of cell membranes, (ii) disruption of protein synthesis by reacting with various amino acids, and (iii) oxidation of nucleic acids and proteins (51). The antibacterial effect of ClO_2_ is mainly due to its destabilization of cell membranes; oxygenated compounds and proteins in cell membranes react with ClO_2_, resulting in the disruption of cellular metabolism (52). These results indicate that the composite film has promising application prospects in antibacterial food packaging.

It has been reported that chlorine dioxide treatment can disrupt the integrity of the cell membrane system of mycobacteria by inducing apoptosis and decreasing the mitochondrial membrane potential. Additionally, ClO_2_ can inhibit mycobacteria by altering cell pH homeostasis and increasing nucleotide leakage and malondialdehyde content (53, 54). In this experiment, chlorine dioxide slow-release films also showed effective inhibition against *Staphylococcus griseus*, and the results are shown in Figure 3C. The antibacterial film had a significant effect on mold inhibition, and as the microcapsule content increased, the diameter of the mycelium gradually decreased. The diameter of mycelium was 8.97 cm in the control group, 4.03 cm after 5%-PVA treatment, 3.12 cm after 10%-PVA treatment, and 1.78 cm after 15%-PVA treatment. The inhibition effect of ClO_2_ on mycorrhizal fungi was weaker than that of bacteria.

#### 3.1.7. Slow-release performance analysis

In this study, we investigated the slow-release performance of antimicrobial films containing 10% microcapsules. The results of our tests are presented in Figure 3F. The films were tested at 23°C and 90% relative humidity and were slow-released for 12 days without any sudden release of chlorine dioxide gas. To analyze the release behavior of the microcapsules, we used a logistic release model, which is characterized by a slow release of microcapsules at the beginning, followed by a rapid release after a certain threshold is reached. The fit equation of the logistic release model was determined to be Q=165.8254–165.9704/(1+(t/7.09403)^0.98691^). We found that the R^2^ value approached 1, indicating a good fit for the model. Our results demonstrate that the logistic release model is suitable for the slow release of microcapsules in fruit packaging. The use of antimicrobial films containing microcapsules can help to prolong the shelf life of fruits by providing continuous protection against harmful microorganisms. Overall, our findings suggest that the use of these films could have significant applications in the food packaging industry.

### 3.2. Effect of PVA films loaded with chlorine dioxide microcapsules on the freshness of blueberries

#### 3.2.1. Effect of PVA films loaded with chlorine dioxide microcapsules on the weight loss rate of blueberries during storage

**Figure 4:**
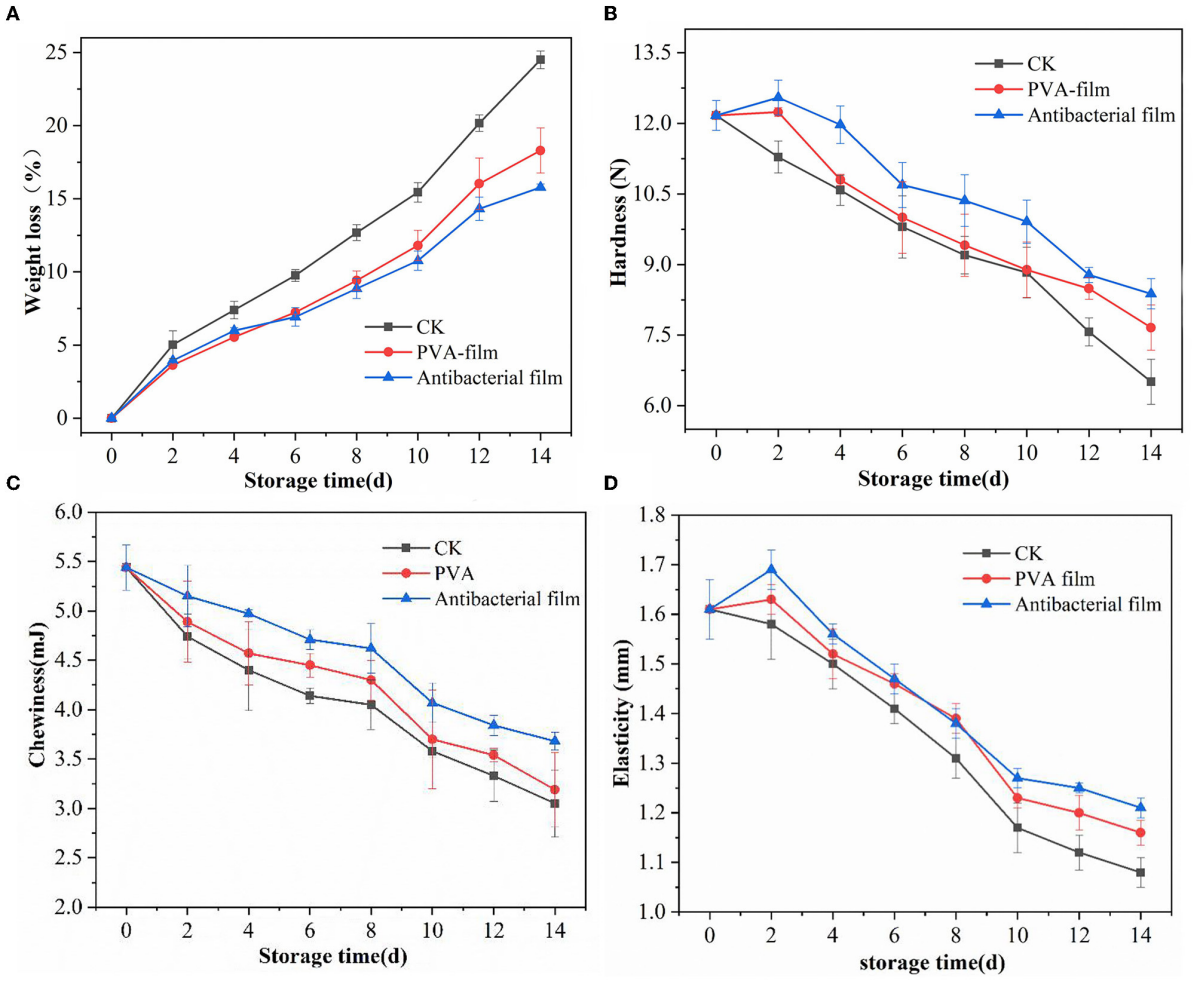
Water weight loss rate of blueberries in each group during storage **(A)**, hardness changes of blueberries in different groups during storage **(B)**, changes in chewiness of blueberries in each group during storage **(C)**, and elasticity of blueberries in different groups during storage **(D)**.

After blueberry fruits are picked, they undergo continuous loss of skin and internal water due to respiration and transpiration, leading to crinkling and weight loss, which affects their quality. Compared with low-temperature storage, blueberries are more active in respiration at room temperature, and the weight loss will be more obvious. The weight loss rate of blueberries is shown in Figure 4A. During storage, all experimental groups showed a similar trend of gradual increase in the weight loss rate with time. Compared with the CK group without treatment, the increasing trend in the weight loss rate of blueberries in both PVA sachets and antibacterial film sachets was relatively slow. On the 14th day of storage, the weight loss rate of blueberries in the CK group was as high as 24.49% and the weight loss rate of blueberries in PVA bags was 18.29%, while the weight loss rate of blueberries in antibacterial film pouches was only 15.78%, which was considerably lower than that of the other two groups, indicating that antibacterial film thinning reduced the water loss of blueberries to a certain extent and had a better effect in controlling the weight loss rate of blueberries.

#### 3.2.2. Textural analysis of PVA films loaded with chlorine dioxide microcapsules during storage of blueberries

TPA is a well-established method for evaluating food products by mimicking the chewing motion of the human mouth and performing reciprocal motion of double compression on food products to obtain a variety of mechanical properties such as hardness, resilience, cohesion, elasticity, stickiness, chewiness, adhesion, and fracture (55). TPA tests were performed on blueberries from all experimental groups during storage. Adhesion is defined for semi-solid foods, and for most fruits, adhesion is zero; hence, the adhesion indicator does not apply to blueberries (56). Therefore, we only compiled data on three important indicators of blueberry quality: hardness, chewiness, and elasticity.

Hardness is an important factor affecting blueberry consumption, and a survey conducted in the US in 2011 pointed out that consumers considered “too soft” as one of the three most common reasons for dissatisfaction after purchasing blueberries (57). The changes in blueberry hardness in each group during storage are shown in Figure 4B. The hardness of blueberries in the CK group showed a gradual decrease, while the hardness of blueberries wrapped in PVA sachets and antibacterial film sachets showed an increasing and then decreasing trend. It is generally believed that the softening of blueberries is related to the change in cell wall components, mainly pectin, cellulose, and hemicellulose, and these three components are linked together by glycosidic and hydrogen bonds to maintain the cell wall in a stable state and thus maintain fruit hardness (58). During storage, the three components degrade and destroy the cell wall structure, causing the fruit to become soft. However, some studies suggest that water loss is the main reason for the change in hardness during storage and that lower water loss does not lead to softening but rather increases the hardness of blueberries to a certain extent (59). This trend was also reflected in this experiment; on the second day of storage, blueberries in PVA sachets and antibacterial sachets had a low water loss, with weight loss rates of 3.63% and 3.93%, respectively, and their hardness were 12.24 N and 12.55 N, respectively, which were higher than the initial hardness. In contrast, the hardness of the CK group did not increase because of the high weight loss rate of 5.01% on day 2 and showed a gradual decrease in hardness with increasing storage time. The hardness of blueberries in the CK group was 6.51 N, while blueberries in PVA sachets had a hardness of 7.66 N, and those in antibacterial film sachets had a hardness of 8.38 N on the 14th day of storage, which was substantially better than that of the other two groups, indicating that the antibacterial film sachets maintained the hardness quality of blueberries better during the storage period and had a certain preservation effect.

The change in the chewiness of blueberries in each group during storage is shown in Figure 4C. The chewiness of blueberries in all three groups gradually decreased with the extension of storage time. After 14 days of storage, the chewiness of blueberries in the CK group decreased by 43%, the chewiness of blueberries protected by the PVA film decreased by 41%, the chewiness of blueberries protected by antibacterial film decreased by 32%, and the chewiness of blueberries protected by the antibacterial film was 3.68 mJ. The antibacterial film delayed the decrease in chewiness to some extent and played a role in maintaining the quality of the blueberries.

Elasticity is the ability of a sample to recover its original height after deformation during the first compression. The greater the elasticity, the lower the softening of the fruit and the better the quality. The changes in the elasticity of the three groups of blueberries during storage are shown in Figure 4D. The elasticity changes were similar to the hardness changes, and the elasticity of blueberries protected by PVA film and antibacterial film initially increased and then decreased, whereas the elasticity of blueberries in the CK group showed a direct decreasing trend. The overall change of elasticity of the three groups of blueberries was fast and then slow, and the decrease of elasticity of blueberries tended to be slow by the 10th day. By the 14th day, the elasticity of blueberries in the CK group decreased by 32%, while the elasticity of the antibacterial film group decreased by 24%, and its elasticity value was 1.21 N. This demonstrates that PVA films containing chlorine dioxide microcapsules can maintain the textural properties of blueberries to some extent.

#### 3.2.3. Changes in soluble solids (TSS) and pH of blueberries

**Figure 5:**
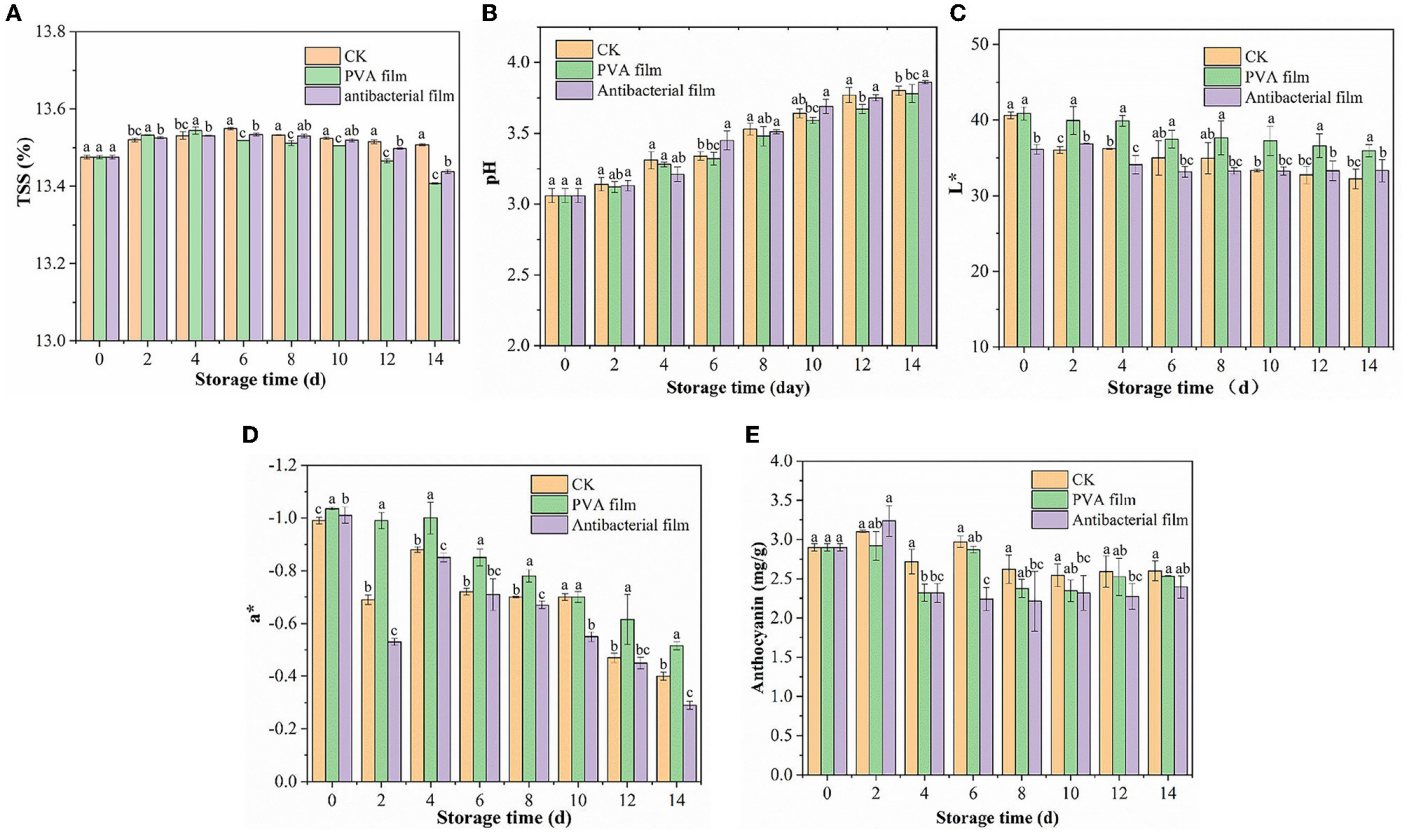
Changes in soluble solids of each group of blueberries during storage **(A)**. The results of pH changes of blueberries in each group during storage **(B)** Changes of L* **(C)** and a* **(D)** values of blueberries in each group during the storage period. Changes in anthocyanin content of blueberries in each group during storage **(E)**.

Soluble solids and pH are basic indicators of sugar and acid content in blueberries. Soluble solids (TSS) are an indicator of fruit ripeness and affect blueberry flavor. During the ripening stage, metabolic reactions are accelerated, leading to an increase in sugar content, which results in an increase in fruit sweetness during storage. The changes in the TSS of the three groups of blueberries during storage are shown in Figure 5A. It was observed that the TSS of the three groups of blueberries showed an overall trend of first increasing and then decreasing. The TSS of all three groups of blueberries reached the highest values at 13.54%, 13.51%, and 13.53%, respectively, when stored until the sixth day. This may be due to the hydrolysis of insoluble polysaccharides into monosaccharides during post-harvest respiration (60). No significant differences in TSS changes in blueberries were observed between the three experimental groups, indicating that ClO_2_ released from the antimicrobial film did not affect TSS, which is consistent with the experimental results of Colgecen (61). However, on the 12th and 14th day of storage, the TSS values of the three groups of blueberries showed significant differences, indicating that the antibacterial film releasing ClO_2_ was effective in maintaining the TSS of blueberries at the later stage of storage.

Acidity can affect the blueberry flavor and is one of the most important factors affecting their consumption. Figure 5B shows the pH changes in the three groups of blueberry samples during storage. The pH of all samples showed an increasing trend owing to the consumption of organic acids during respiration. The pH of blueberries in CK, PVA film, and antimicrobial film increased from 3.06 to 3.8, 3.78, and 3.86, respectively, after storage for up to 14 d. Comparing the pH changes of the three experimental groups, we did not observe significant differences. Therefore, PVA films loaded with chlorine dioxide microcapsules did not substantially affect the TSS and pH of blueberries during storage.

#### 3.2.4. Effect of PVA films loaded with chlorine dioxide microcapsules on blueberry color difference and anthocyanin content during blueberry storage

Color is an important attribute for consumers to perceive the quality of blueberries, and surface color is a crucial factor that affects the visual appearance of fresh blueberry fruit. L^*^ and a^*^ values are the main indicators used to assess the color change of blueberries. The L^*^ value is the brightness value of the blueberry fruit and a^*^ is the red and green values. For a positive a^*^, the larger the value, the greater the increase in redness; and for a negative a^*^, the larger the value, the greater the greenness. The three groups of blueberries changed in color during storage, as shown in Figures 5C, D. The L^*^ values of the CK, PVA film, and antibacterial film groups were 40.60, 40.88, and 36.13, respectively, on day 0 of storage, and decreased to 32.23, 35.95, and 33.32, respectively, on day 14. It means that the three groups of blueberries are significantly different in L^*^. This indicates that with an increase in storage time, the fruit gradually aged, and the color gradually dulled. The brightness of the fruit also gradually decreased. However, the antibacterial film group decreased more slowly, with a 7.77% decrease from the initial L^*^ value, while the CK group decreased by 20.63%. The a^*^ values were all negative and showed a fluctuating upward trend; however, the changes were not obvious. The a^*^ values of the CK, PVA film, and antibacterial film group were −0.99, −1.035, and −1.01, respectively, at the beginning of storage and increased to −0.4, –.515, and −0.29, respectively, by 14 days. This may be because the skin of the blueberry fruit was exposed as the frost on the skin of the fruit fell off with the extension of storage time, and the color of the blueberry fruit deepened to red due to aging.

Anthocyanins are water-soluble glycosides that are beneficial in a variety of chronic diseases, including cardiovascular diseases, neurodegenerative diseases, diabetes, and cancer (62). Figure 5E shows the changes in anthocyanins in the three groups of blueberries during storage. The three groups of blueberries showed an initial rising trend followed by a downward fluctuation. The increase in anthocyanin content is due to the continuous maturation of blueberries, where the synthesis of anthocyanins is greater than their consumption of blueberries at the beginning of storage, resulting in cumulative increases. This may be because, with the extension of storage time, the ClO2 released from the antibacterial film gradually increased in the packing tape, which oxidized some of the anthocyanins in the blueberry skin. After all, the anthocyanin content of the CK group was 2.6 mg/g FW and that of the PVA film group was 2.53 mg/g FW. Anthocyanins are related to fruit color, which confirms why blueberries packed with the antimicrobial film had the highest a^*^ value after 14 days. However, the effect of ClO_2_ was not substantial, and there was no visible difference in the appearance of blueberries, with only a slight decrease in anthocyanin content.

#### 3.2.5. Moisture content and distribution of blueberries in PVA films loaded with chlorine dioxide microcapsules during blueberry storage

**Figure 6:**
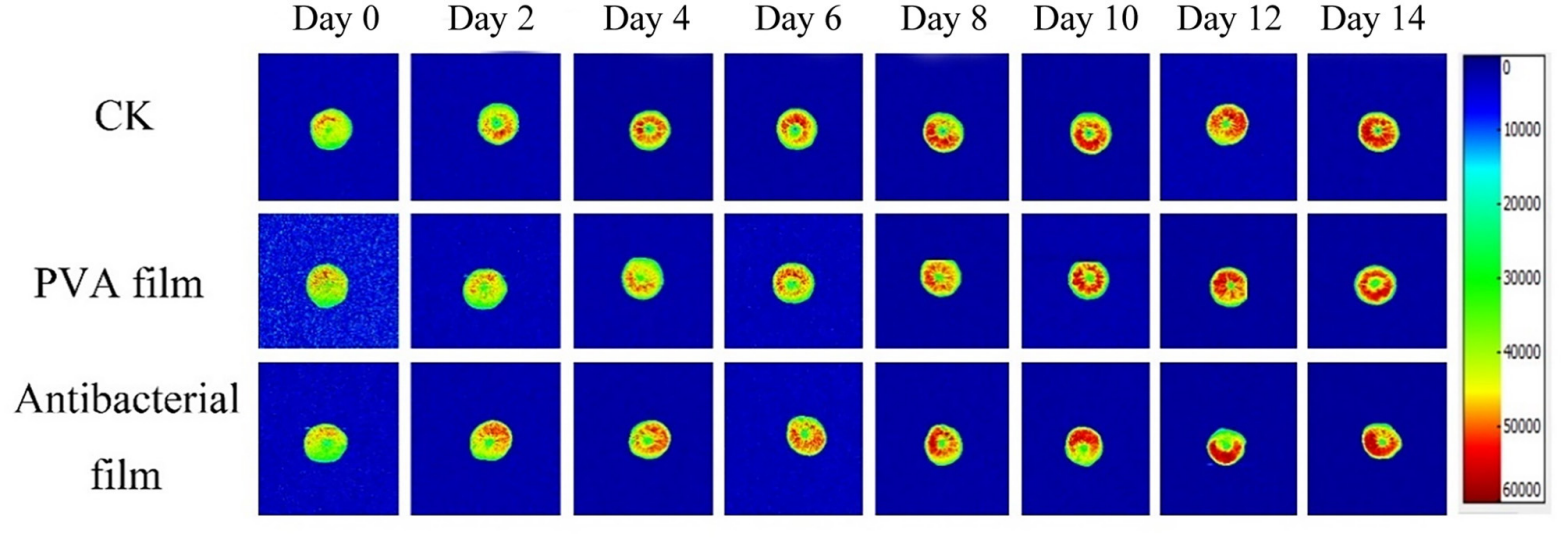
Pseudo-color image of blueberry MRI.

**Figure 7:**
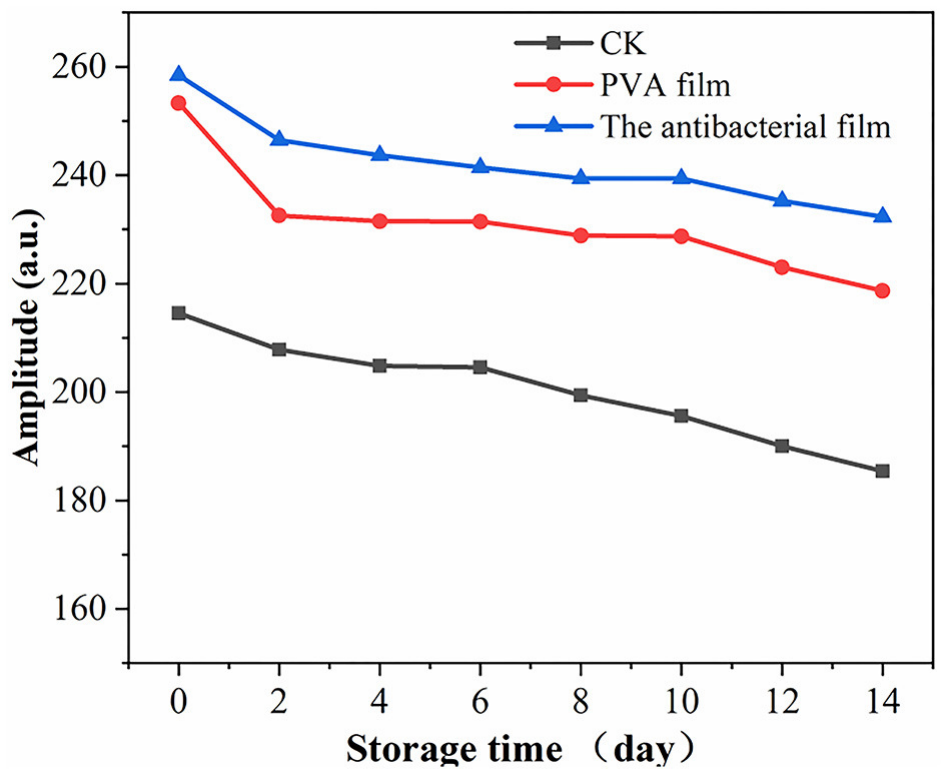
Overall moisture change of blueberries during storage.

Moisture is a major component of food, and its content, distribution, fluidity, and changes directly affect the appearance, structure, rheological properties, stability, sensitivity to microorganisms, and shelf life of food. There are three different states of water in blueberries: bound water present in the cell wall, fixed water present in the cytoplasm, and free water present in vesicles. Nuclear magnetic resonance imaging (MRI) can be used to directly observe water distribution in blueberries. The different colors in the pseudo-color image represent the different water contents in the sample; the redder the color, the higher the density of water molecules in a given region (63, 64). The MRI detection of the three-figure blueberries is shown in Figure 6. The color distribution of the blueberry pseudo-color map at the beginning of storage was uniform, indicating that the moisture distribution of fresh blueberries was uniform. However, with time, the blueberry pseudo-color map appeared red locally, indicating that blueberry moisture migrated during storage. The overall moisture change of blueberries during storage is shown in Figure 7. Although the local moisture content of blueberries increased, the overall moisture content decreased with the extension of storage time. The lowest moisture loss in blueberries in the antimicrobial film group was observed after 14 days of storage, which is consistent with the results of the weight loss rate.

#### 3.2.6. Changes in the appearance of blueberries during storage in PVA films loaded with chlorine dioxide microcapsules

**Figure 8:**
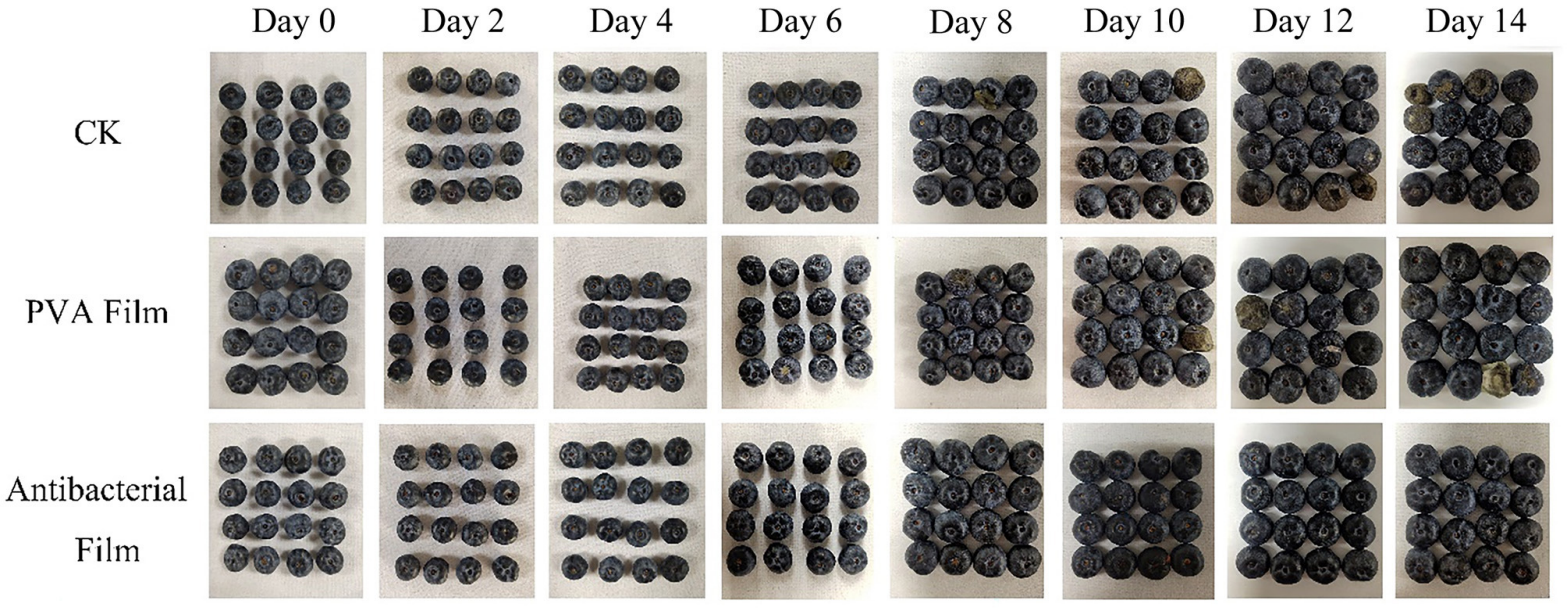
Changes in the appearance of blueberries during storage.

Blueberries are usually packed in small boxes; hence, pressing them to feel their hardness and judge their freshness is not allowed in the market. The appearance of blueberries can also help us judge their quality directly, as dull color, denting, or rotting by fungal infection are all signs of deterioration. Therefore, we recorded the sensory changes in the three groups of blueberries during storage, as shown in Figure 8. At the beginning of storage, all blueberries had a good appearance and were shiny. However, by day 6, the blueberries in the CK group were contaminated with fungi and showed decay and deterioration, and in the PVA film group, mold growth was observed at the tips, but not as severe as in the CK group, while all groups showed water loss and wrinkling from day 6 onward. With the extension of storage time, fungal contamination gradually expanded and spread from the fruit tips to the whole fruit, and fungal contamination occurred only on one blueberry fruit at the beginning, and then several blueberry fruits showed fungal contamination decay. However, in the antimicrobial film group, no mold was observed from the beginning to the end, which indicates that the antimicrobial film not only has a good effect on the *in vitro* antimicrobial test but can also play a role in protecting blueberry fruits from fungal contamination in actual preservation applications. Furthermore, the blueberries in the CK group had the most serious water loss and wrinkling in the late storage period, followed by the PVA film group. Only the blueberries in the antimicrobial film group maintained a relatively good appearance, with no fungal contamination and no obvious water loss and wrinkling of the peel, which is consistent with the weight loss rate. Therefore, based on the changes observed in blueberry morphology, it was concluded that PVA films loaded with ClO_2_ microcapsules may extend the shelf life of blueberries during storage, and most factors could be better maintained at a better level.

In this study, we prepared PVA films containing ClO_2_ microcapsules using microencapsulation technology, characterized the antibacterial films, and investigated the performance of PVA films containing ClO_2_ microcapsules for blueberry preservation. When the microcapsules were added at 10%, the film fracture strength increased by 180.81% compared with the pure PVA film, the water vapor transmission rate decreased by 22% compared with that of the pure PVA film, and the UV barrier performance was also improved. The UV transmission rate at 280 nm decreased by 57.37%, and the thermal stability of the antimicrobial film was also improved. Additionally, the antimicrobial film containing ClO_2_ microcapsules had a good antibacterial effect, with 93.69% inhibition against *Listeria monocytogenes* and 95.55% inhibition against *Escherichia coli*, and successfully inhibited the mycelial growth of *Staphylococcus griseus*. The slow-release time of the antimicrobial film containing ClO_2_ microcapsules was 12 days at 23°C and 90% RH, and there were no issues related to the ClO_2_ gas spike during the slow-release period.

## 4. Conclusion

In this study, it was found that the best overall performance of the self-releasing film was achieved at 10% microcapsule content. The mechanical properties of the film were significantly improved, with the fracture strength increasing to 66.74 MPa. The thermal stability was also improved, with T10% increasing to 278°C. The barrier properties of the film were also improved, with the water vapor barrier decreasing to 361.18 g/m^2^·24 h. At the same time, the barrier performance of the film was improved, and the water vapor barrier decreased to 361.18 g/m^2^·24 h. The film had a good antibacterial effect, with 93.69% for *Listeria monocytogenes* and 95.55% for *Escherichia coli*, and had successfully inhibited the mycelial growth of Staphylococcus griseus. In the application of PVA films containing ClO_2_ microcapsules for blueberry preservation, the ClO_2_ self-release antimicrobial film group was able to slow the quality deterioration of blueberries. During the 14-day storage cycle, no mold contamination was observed in the ClO_2_ self-releasing film group. The weight loss rate was 24.49% in the CK group and 15.78% in the ClO_2_ self-releasing film group by the 14th day of storage while maintaining the hardness of the blueberries. Blueberries in the antimicrobial film group had higher anthocyanin accumulation of 3.23 mg/g during storage. Furthermore, local moisture migration was observed in the blueberries, and the reduction in moisture content was relatively delayed in the antimicrobial film group. These results suggest that PVA films containing ClO_2_ microcapsules are promising materials for future fruit and vegetable packaging.

## Data availability statement

The original contributions presented in the study are included in the article/supplementary material, further inquiries can be directed to the corresponding author.

## Author contributions

CH and HS conceived and designed the experiments. ZC, JA, and RL performed the experiments. YZ, RL, and HH analyzed the data. ZC, JA, HH, and RL contributed reagents, materials, and analysis tools. YZ, HS, and ZC wrote the manuscript. CH and HH edited the manuscript. All authors contributed to the article and approved the submitted version.
