# Supplementary figures and images for: Polyvinyl alcohol film with chlorine dioxide microcapsules can be used for blueberry preservation by slow-release of chlorine dioxide gas
Source: Front Nutr. 2023 Apr 18;10:1177950. doi: 10.3389/fnut.2023.1177950 (PMC10151673; doi:10.3389/fnut.2023.1177950)

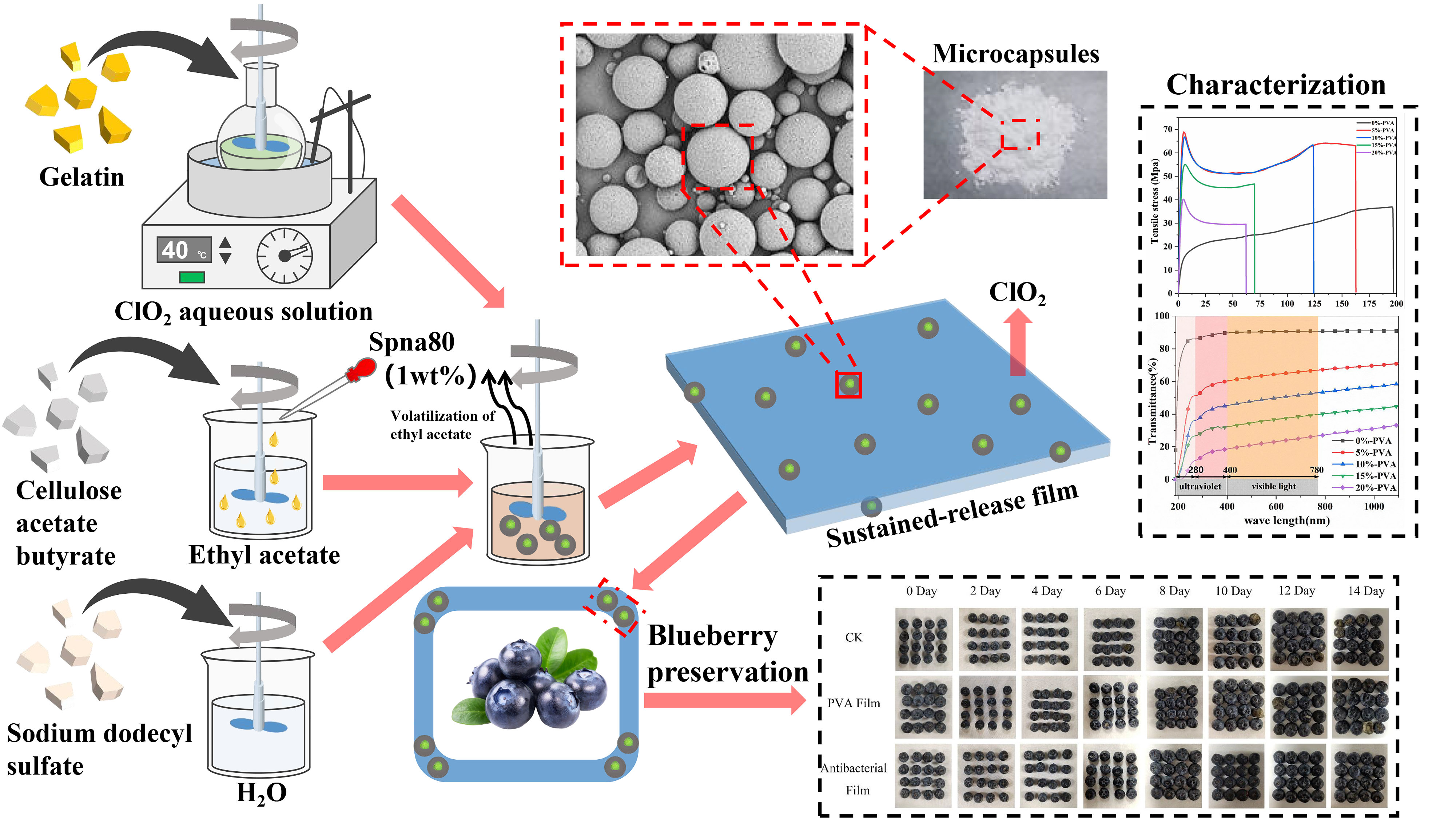

Supplement: Supplementary file 1 [file Image_1.TIF]
